# Inhibitory Effects of Lactobionic Acid on Biofilm Formation and Virulence of *Staphylococcus aureus*

**DOI:** 10.3390/foods13172781

**Published:** 2024-08-31

**Authors:** Shimo Kang, Yahui Yang, Wanwan Hou, Yan Zheng

**Affiliations:** 1 College of Food Science, Shenyang Agricultural University, Shenyang 110161, China; shimokang@sinh.ac.cn (S.K.);; 2 CAS Engineering Laboratory for Nutrition, Shanghai Institute of Nutrition and Health, University of Chinese Academy of Sciences, Chinese Academy of Sciences, Shanghai 200031, China; 3 Department of Food Science & Technology, School of Agriculture and Biology, State Key Lab of Microbial Metabolism, Shanghai Jiao Tong University, Shanghai 200240, China

**Keywords:** *Staphylococcus aureus*, lactobionic acid, biofilm formation, virulence

## Abstract

*Staphylococcus aureus* biofilm is a common bio-contaminant source that leads to food cross-contamination and foodborne disease outbreaks. Hence, there is a need for searching novel antibiofilm agents with potential anti-virulence properties to control *S. aureus* contamination and infections in food systems. In this study, the antibiofilm effects of lactobionic acid (LBA) against *S. aureus* and its influence on virulence were explored. The minimum inhibition concentration of LBA on *S. aureus* was 8 mg/mL. Viable count and crystal violet assays revealed that LBA inhibited and inactivated *S. aureus* biofilms. Microscopic observations further confirmed the antibiofilm activity of LBA on *S. aureus* that disrupted the biofilm architecture and inactivated the viable cells in biofilms. Moreover, LBA decreased the release of extracellular DNA (eDNA) and extracellular polysaccharide (EPS) in *S. aureus* biofilms. LBA suppressed biofilm formation by intervening metabolic activity and reduced virulence secretion by repressing the hemolytic activity of *S. aureus*. Furthermore, LBA altered the expressions of biofilm- and virulence-related genes in *S. aureus*, further confirming that LBA suppressed biofilm formation and reduced the virulence secretion of *S. aureus*. The results suggest that LBA might be useful in preventing and controlling biofilm formation and the virulence of *S. aureus* to ensure food safety.

## 1. Introduction

Microbial biofilms are the main factor causing food cross-contamination and foodborne disease transmission, which seriously threaten food safety and cause numerous economic losses [1]. The World Health Organization (WHO) reported that more than sixty percent of foodborne outbreaks as well as eighty percent of clinical bacterial infections are connected with biofilm formation [2]. Biofilms are many microorganisms aggregated film-like substances formed when microorganisms irreversibly adhere to abiotic or living surfaces [3]. They are capsuled in a matrix of extracellular polymeric substances such as extracellular DNA (eDNA), extracellular polysaccharides (EPS), and extracellular proteins secreted by themselves [3], which possess a complex and compact three-dimensional structure, which can generate a strong barrier effect, providing physical and chemical protection for the bacterial population, resisting antibiotic attacks and evading host immunity and other unfavorable conditions [4,5]. The environment of food processing plants has many conditions beneficial for bacterial adhesion and biofilm formation such as moisture, nutrients, etc. Most common foodborne spoilage and pathogen bacteria can adhere to the surface of food, most processing equipment, and almost all environmental conditions to form biofilms that cause a bacterial burden on the complete food system, leading to food deteriorates and shortening shelf life, further increasing the risk of outbreaks of foodborne diseases [6,7]. Hence, how to control and remove the biofilm pollution of pathogens is of crucial importance for ensuring food safety.

*Staphylococcus aureus* (*S. aureus*) is a common foodborne pathogen that readily forms biofilms onto food and food-contact superficials to cause food contamination, greatly increasing the risk of foodborne disease outbreaks [8]. The pathogenicity of *S. aureus* is ascribed to various virulence factors such as α-hemolysin, protein A, etc. [9]. Furthermore, *S. aureus* multiplying in food can also produce *S. aureus* enterotoxin, which can cause severe gastroenteritis after ingestion, causing nausea, vomiting, abdominal cramps, and other symptoms [10]. *S. aureus* has become second only to *Salmonella*, and *Vibrio parahaemolyticus* causes bacterial food poisoning as the third most important pathogen, and its biofilm state is the main cause of *Staphylococcal* foodborne illness outbreaks [11]. Even if sanitizers such as benzalkonium chloride and hydrogen peroxides are used regularly for cleaning and disinfecting, *S. aureus* can still survive, resulting in the beginning of drug-resistant *S. aureus* [6]. Therefore, it is essential to find novel antibiofilm agents with potential anti-virulence properties to contribute to decreasing drug resistance. 

Regarding lactobionic acid (LBA), an organic acid naturally found in Caspian Sea yogurt, its calcium salt has been approved as a food additive by the FDA [12]. In the food industry, LBA is known for its broad applications, e.g., as a thickener, acidifier, moisturizer, gelling, and metal chelating agent, but it also has health benefits, such as through its antimicrobial, anti-obesity, antioxidant, and prebiotics effects, etc. [13]. Previously, we found that LBA showed excellent antibacterial and antibiofilm abilities to combat *S. aureus*, then further revealed the antibacterial mechanism of LBA against *S. aureus* at the cell and protein levels [14,15]. However, little is known about the inhibitory effect of LBA on *S. aureus* biofilm formation and virulence. Hence, in this study, we evaluated the inhibitory activity and the possible mode of action of LBA on *S. aureus* biofilm and the influence on virulence by adopting an in vitro model. Microscopic morphological changes of *S. aureus* biofilms were observed by scanning electron microscopy (SEM) and confocal laser scanning microscopy (CLSM). Furthermore, the metabolic and hemolysis activities of LBA on *S. aureus*, as well as the mRNA expression levels of biofilm- and virulence-related genes in *S. aureus*, were also determined. The findings will accelerate the potential application of LBA as a novel antibiofilm agent with potential anti-virulence properties. 

## 2. Materials and Methods

### 2.1. Reagents, Bacterial Strains, and Culture Conditions

The *S. aureus* ATCC 25923 strain used in this study was obtained from the American Type Culture Collection (ATCC) that isolated it from a clinical setting and was stored in our laboratory at –80 °C before use. Bacteria was cultured on trypticase soy agar (TSA) medium (Land Bridge, Beijing, China) at 37 °C for 24 h. A single colony, which was incubated with shaking at 160 rpm for 18 h at 37 °C in 30 mL of sterile tryptic soy (TSB) broth (Land Bridge, Beijing, China), had a pellet obtained by centrifugation at 8000× *g* for 10 min at 4 °C and by rinsing twice with sterile TSB. Then, the cell pellet was resuspended in sterile TSB to achieve a final OD 600 value of 0.5 and was used in subsequent assays. LBA (≥98% purity, CAS: 96-82-2) was bought from Sigma (St. Louis, MO, USA). All other reagents were bought from Sinopharm Group Chemical Reagent Co., Ltd. (Shanghai, China). 

### 2.2. Minimum Inhibitory Concentration (MIC)

MIC determination was measured by referring to the Clinical and Laboratory Standards Institute (CLSI) broth microplate assay guidelines that we used in our previous work [14]. Briefly, LBA was first resolved in sterilized water, then gradually diluted with sterilized water. In a 96-well microtiter plate containing 100 µL of TSB broth, serial two-fold dilutions of LBA at concentrations ranging from 128 to 0.5 mg/mL were performed. Each well contained 2 µL suspensions at a concentration of 0.5 at OD_600_ and were cultured for 24 h at 37 °C. The MIC value was subjected to the minimal concentration of LBA that suppressed visible growth.

### 2.3. Crystal Violet Assay

Biofilm formation assay was conducted at diverse LBA concentrations by employing 96-well microtiter plates from our prior work [16]. Briefly, cell suspensions in TSB broth to give an OD 600 of 0.5 were cultured with or without LBA at the final concentration of 0.25 × MIC, 0.5 × MIC, and 1 × MIC at 37 °C for 24 h. The attached biofilms were resolved in 100% ethanol after being stained with 0.1% crystal violet. The total amount of biofilm formation was quantified by determining the absorbance at 595 nm. 

### 2.4. Viable Count Assay

The viability count on the *S. aureus* biofilm cells was conducted as previously reported with slight modifications [17]. *S. aureus* biofilms were generated on coverslips (1 cm × 1 cm) placed on 24-well microtiter plates. An amount of 0.6 mL of sterile 2% agar solution was added to each well, and after the agar had solidified, sterile coverslips (1 cm × 1 cm) were inserted into the center of the solidified agar of each well. Then, 0.1 mL cell suspensions and 1.9 mL TSB medium were added to each well, incubated with or without LBA at the final concentrations of 0.25 × MIC, 0.5 × MIC, and 1 × MIC at 37 °C for 1 d, 3 d, and 5 d, respectively. Then, the coverslips were gently removed and rinsed with PBS to erase loosely adhered cells, then gently transferred to the test tubes containing equal PBS for ultrasonic treatment, such that the adhesive substances on the coverslips could be completely dissolved in the PBS to form biofilm suspensions. The biofilm suspensions from appropriate dilutions were spread on TSB agar plates and cultured at 37 °C for 24–48 h, then we counted the colonies numbers.

### 2.5. Microscopic Visualization

#### 2.5.1. Visualization by SEM

The changes of *S. aureus* biofilms in morphology were observed by SEM as previously described [10]. The biofilms were cultured shown above (Section 2.4). Coverslips were gently removed after 72 h of culturing, rinsed with PBS, fixed at 4 °C with 2.5% glutaraldehyde overnight, and dehydrated using gradient ethanol. Then, undergoing critical-point drying and platinum sputter-coating, the images of biofilm cells sustained SEM (Zeiss EVO-LS10, Cambridge, UK) at a 20 kV accelerated voltage and 20,000× magnification. 

#### 2.5.2. Visualization by CLSM

The disruption situation of biofilms after LBA treatment was observed by CLSM as previously reported [18]. The biofilms were cultured as mentioned earlier (Section 2.5.1). After rinsing with PBS, stained with a LIVE/DEAD BacLight bacterial viability kit (Solarbio, Beijing, China), which contains equal volumes of SYTO 9 and propidium iodide (PI) stains. Then, incubated away from light at 25 °C for 30 min, the stained biofilms were observed with a CLSM after rinsing with sterile filtered water. Two stains were separately imaged first and then scanned on each biofilm sample. The excitation/emission of PI and SYTO 9 were 555 nm/more than 575 nm and 488 nm/less than 550 nm, separately. Images were captured and processed using ZEN 2010 software for visualization.

### 2.6. Determination of Extracellular Polymeric Matrix 

#### 2.6.1. EPS Content

The colorimetric method of anthrone sulfuric acid was used to measure the EPS content in biofilms as stated before [19]. Firstly, the standard glucose solutions with different concentrations (0, 20, 40, 40, 80, and 100 μg/mL) were completely mixed with 80% sulfuric acid (containing 5 mg anthrone) of 5 mL, respectively, boiled in a bath for 15 min, then ice bathed for 15 min. Then, the standard glucose curve was established by determining the absorbance at a 625 nm wavelength. Secondly, the biofilms were incubated as described above (Section 2.4). Coverslips were gently removed after 24 h of culturing, rinsed with PBS, then gently transferred into the test tubes containing 2 mL PBS for ultrasonic treatment to form biofilm suspensions. Then, the resulting supernatants were collected by centrifugation for 10 min at 9500× *g*, then mixed thoroughly with 80% sulfuric acid (containing 5 mg anthrone) of 5 mL, boiled in a bath for 15 min, then ice bathed for 15 min. Lastly, the OD 625 values were recorded, then we calculated the EPS concentrations by the standard glucose curve. 

#### 2.6.2. eDNA Content

The biofilms were incubated as stated before (Section 2.3), in which the eDNA in the biofilms was determined using the spectrophotometric method [20]. After PBS washing, each well contained 0.5 M EDTA and was incubated for 1 h at 4 °C, then cells were resuspended by adding 700 μL of 50 mM TEN buffer to each well. The supernatant was collected after centrifugation at 18,000× *g* for 5 min, then shifted into another tube containing 300 μL of TE buffer. Next, the binding buffer and the mixture were equally added to adsorption columns, then the eDNA was gathering by centrifugation for 1 min at 18,000× *g* and washed thrice by wash buffer. Then, we used a NanoDrop spectrophotometer (Thermo Fisher Scientific, Waltham, MA, USA) to quantify the extracted eDNA, which dissolved in sterile water.

### 2.7. Biofilm Metabolic Activity

A 2,3-bis(2-methoxy-4-nitro-5-sulfophenyl)-2H-tetrazolium-5-carboxanilide sodium salt (XTT) reduction assay was used to measure the metabolic activity [21]. The biofilms were cultured as shown above (Section 2.3). Then, the precipitates were collected by centrifugation at 1000× *g* for 5 min after rinsing with PBS. Each well of the 96-well microtiter plate containing a 200 μL mixture included a final concentration of 150 μg/mL of XTT and 10 μg/mL of phenazine methanesulfonate (PMS), incubated away from light at 37 °C for 3 h with 120 r/min. The metabolic activity was determined by reading the OD 490 value in each well using a microplate reader. 

### 2.8. Hemolysis Measurement

A hemolysis assay was measured with red blood cells (RBCs) in liquid media [22]. The diluted (1:100) overnight-grown cultures (5 mL) were incubated with LBA at the final concentrations of 0.25 × MIC, 0.5 × MIC, and 1 × MIC, respectively, and incubated with shaking at 250 rpm at 35 °C for 24 h. Next, 1 mL of 3.3% sheep RBCs was mixed with 100 μL of overnight cultures and incubated with shaking at 250 rpm at 35 °C for 1 h. The collected supernatants centrifugated at 10,000×g for 10 min were determined at the absorbance at 543 nm.

### 2.9. RNA Isolation and qRT-PCR

Biofilms were collected as previously mentioned (Section 2.3). A TIANamp RNAprep pure Cell/Bacteria Kit (Tiangen, Beijing, China) was used to extract the total RNA. The relative transcriptional levels of icaA, icaR, agrA, sigB, and hla in S.aureus biofilm cells treated without and with 1 × MIC LBA were measured using qRT-PCR as described previously [23]. cDNA was quantified using the AceQ qPCR SYBR Green Master Mix (Jizhen Biology, Shanghai, China) in a CFX96 qPCR system (Bio-rad). The comparative threshold cycle (Ct) method was utilized to calculate the mRNA levels of target genes. Samples were measured in triplicate in three independent experiments. 16sRNA was performed as the normalization control. The primers are listed in Table 1. 

### 2.10. Statistical Analysis

Experiments were conducted in triplicate. Data were analyzed with a variance and Duncan test with SPSS software (version 22) and presented as mean ± SDs (*n* = 3). *p* < 0.05 was expressed as statistically significant.

## 3. Results and Discussion

### 3.1. Effect of LBA on Biofilm Formation of S. aureus

In this study, we employed the micro-broth dilution method to measure the MIC of LBA against *S. aureus*; the MIC value was 8 mg/mL. It is noteworthy that the MIC value obtained in this study was in contrast to our previous work; the previous MIC values for LBA against both the SJTUF21564 and N315 strains were 12.5 mg/mL [16], which may have been due to their strong biofilm-producing abilities. The observed decrease in the MIC for ATCC 25923 may be attributed to weaker biofilm-producing abilities compared to N315 and SJTUF21564, as well as LBA variations in the purity sourced from diverse producers. 

**Figure 1:**
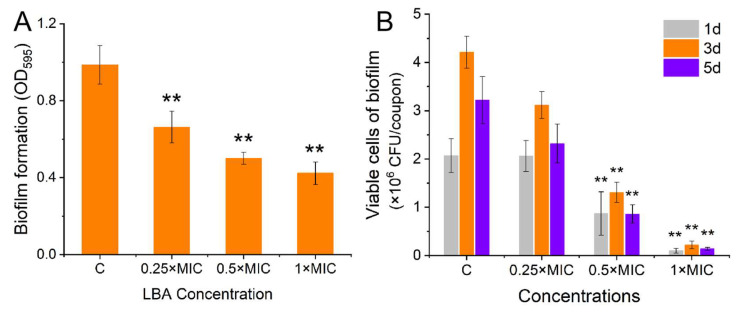
Effect of LBA on *S. aureus* biofilm formation (**A**) and the viable cells in biofilms (**B**). ** *p* < 0.01, compared with control.

Further, the biofilm formation of S. aureus and viable cells in S. aureus biofilms treated with LBA at diverse concentrations were drawn. The results indicated that LBA exhibited antibiofilm activities that were concentration-dependent against S. aureus. A dose-dependent decline trend in biofilm formation and viable cells in biofilm were observed (Figure 1). LBA at the final concentrations of 0.25 × MIC, 0.5 × MIC, and 1 × MIC significantly suppressed *S. aureus* biofilm formation, with reductions of 32%, 48%, and 56% (Figure 1A), respectively, compared with those of the control (*p* < 0.01). Moreover, the impact of the LBA-inhibited *S. aureus* biofilm formation was evident by evaluating the binding of crystal violet to biofilm cells cultivated on the microplate. Similar studies were reported in other organic acids like citric acid [24], Boswellic acid [25], and shikimic acid [26]. When treated with 0.5 × MIC and 1 × MIC, the LBA significantly (*p* < 0.01) decreased the viable cells in *S. aureus* biofilms in different incubation times (Figure 1B). No significant decrease in the viable cells of the biofilms was observed at the final concentration of 0.25 × MIC. The results demonstrated that LBA exhibited a significant (*p* < 0.01) antibiofilm activity in *S. aureus* that could inactivate cells in biofilms. Other organic acids could inactivate cells within biofilms, too [27,28]. 

### 3.2. Micromorphological observation

**Figure 2:**
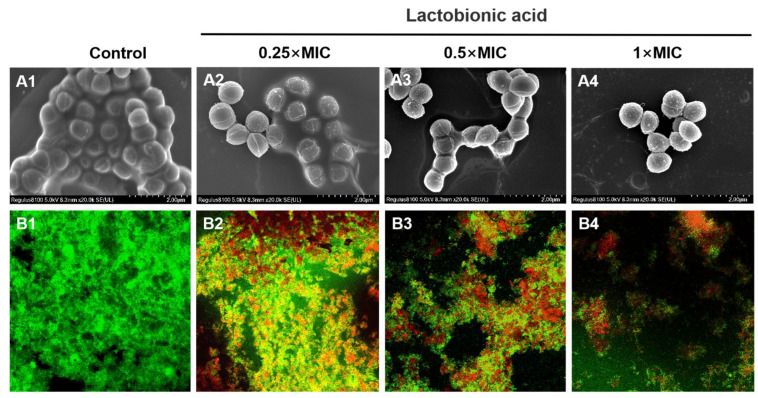
Microscopic visualization of *S. aureus* biofilms treated with diverse LBA concentrations. (**A1**–**A4**) SEM images at 20,000× magnification. (**B1**–**B4**) CLMS images at 20× magnification.

Biofilm formation begins when free-floating microorganisms attach to the surface and build a distinctive biofilm architecture [29]. The three-dimensional architecture built by colony aggregation and accumulation is a prominent feature in bacterial biofilms [30]. SEM and CLMS revealed the morphology of *S. aureus* biofilm and the bacterial activity in *S. aureus* biofilm on the coverslips after LBA treatment. From the control group of the SEM images (20,000× magnification), the bacterial cells were covered by the visible multi-layered biofilm (Figure 2A1). The surface was uniform and densely packed with biofilm, and a dense three-dimensional structure was formed locally, showing the mucus-like substance between the bacteria (Figure 2A1). As the LBA treatment concentration increased, the adhesion substances gradually decreased and the multilayer structure was dispersed (Figure 2A2–A4), clearly showing a concentration-dependent modification in the *S. aureus* biofilm micro-architecture. The SEM observations indicated that LBA inhibited biofilm formation by reducing cell adhesion and destroying the micro-architecture of *S. aureus* biofilm. In addition, SEM observations further revealed that the release of EPS may have been reduced, which is essential for protection or surface attachment caused by the absence of microcolonies. Similar impacts of LBA were also seen for *Vibrio parahaemolyticus* [28] and *Salmonella typhimurium* [31]. 

In the subsequent investigation, CLMS images (20× magnification) were performed to visualize the inactivation of *S. aureus* biofilm cells after LBA treatment (Figure 2B). *S. aureus* biofilm cells were stained with a Calcein-AM/PI LIVE/DEAD fluorescent kit, and both could be observed under fluorescence excitation after binding to bacterial DNA. Among these, living cells showed green fluorescence while dead or damaged cells showed red fluorescence. In the control group, *S. aureus* biofilms exhibited strong green fluorescence, cell clusters relatively dense, and aggregations, indicating that most *S. aureus* cells in biofilm were alive (Figure 2B1). However, red fluorescence gradually increased and green fluorescence gradually reduced as the LBA dose increased (Figure 2B2–B4), showing that LBA inactivated *S. aureus* cells in biofilms in a concentration-dependent manner. Further, the micro-architecture of *S. aureus* biofilm exhibited loose microcolonies, uneven and less dense after LBA treatment. The CLMS observations suggested that LBA could break the preformed *S. aureus* biofilms by slackening its microcolonies, leading to a collapse of the micro-architecture of *S. aureus* biofilm [18]. This aligned with previous studies indicating that numerous antibiofilm agents can break intricate biofilm micro-architectures and loosen microcolonies [32].

### 3.3. Effect of LBA on the Releases of the Extracellular Polymeric Matrix in S. aureus Biofilm

**Figure 3:**
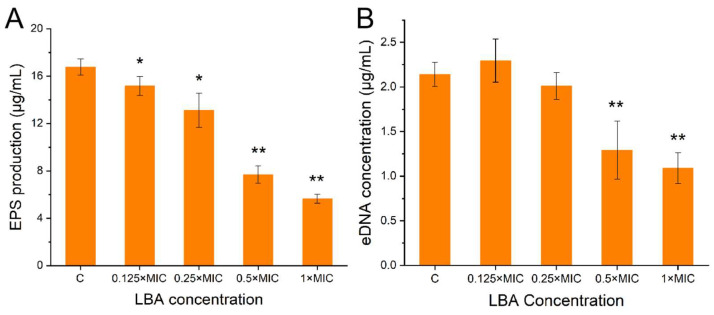
Effect of LBA on the release of EPS (**A**) and eDNA (**B**) of *S. aureus* biofilm cells. * *p* < 0.05, ** *p* < 0.01, compared with control.

The three-dimensional architecture of *S. aureus* biofilms is predominantly attributed to the encapsulation that extracellular matrixes often produce by themselves [33]. EPS and eDNA are major elements of biofilm structure, vital at the bacterial adhesion and proliferation stage, respectively [34,35]. Hence, the effects of LBA on the release of EPS and eDNA in *S. aureus* biofilm were examined. We measured the polysaccharide content to evaluate the impact of LBA on the release of EPS. From Figure 3A, LBA significantly decreased the EPS production in the *S. aureus* biofilms in a concentration-dependent manner (*p* < 0.05), indicating that LBA suppressed the EPS release by *S. aureus*. When treated with LBA at the concentration of 1 × MIC, the EPS production in *S. aureus* biofilm was 5.65 μg/mL with a decrease of 66.31%. Previous studies have reported a similar phenomenon in which the antibiofilm agents could reduce EPS production by *S. aureus* [36]. 

eDNA is a molecular vital matrix that is important for electrostatic forces in the adsorption of bacteria to the surface of objects and the adhesion in acid–base interactions between bacteria, which is essential for stabilizing the mature biofilm structure [37,38]. The results of the release of eDNA in *S. aureus* biofilm are shown in Figure 3B. LBA slightly increased and decreased the eDNA productions in *S. aureus* biofilms after treatment at the final concentration of 0.125 × MIC and 0.25 × MIC, respectively. While treated with 0.5 × MIC and 1 × MIC, LBA significantly reduced the release of eDNA with a decrease of 39.72% and 49.10% (*p* < 0.01), respectively (Figure 3B). This indicated that LBA suppressed the EPS release by *S. aureus*. Many antibiofilm agents are similarly reported to suppress the formation of *S. aureus* biofilm by reducing eDNA release [18]. The above results indicated that LBA suppressed biofilm formation by reducing eDNA and EPS productions by *S. aureus*, further breaking the micro-architecture. SEM and CLMS images showed that the extracellular matrix around the cells of *S. aureus* biofilm decreased with increasing LBA concentrations, further supporting the conclusion.

### 3.4. Effect of LBA on the Biofilm Metabolic Activity and Hemolysis Activity of S. aureus 

**Figure 4:**
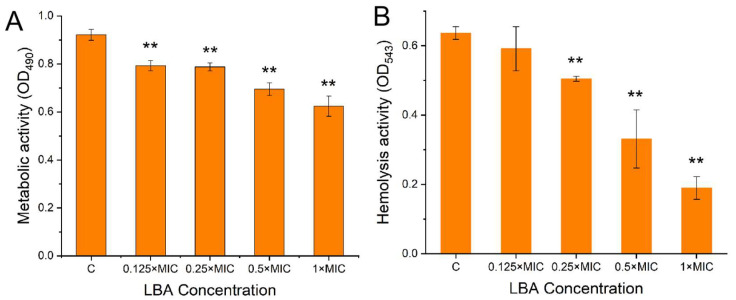
Effect of LBA on the biofilm metabolic activity (**A**) and hemolysis activity (**B**) of *S. aureus*. ** *p* < 0.01, compared with control.

Cellular metabolic activity is one of the crucial elements in the adhesion and formation of biofilms. Many antibiofilm agents prevent bacterial biofilm formation while reducing bacterial biofilms’ cellular metabolic activity [39]. We used the XTT method to explore the metabolic activity of cells in *S. aureus* biofilms, which is efficient and commonly used. Metabolically active cells reduce the XTT to orange water-soluble formazan [21], hence a positive correlation between the XTT releases and cell metabolic activity. From Figure 4A, LBA significantly (*p* < 0.01) decreased the metabolic activity of *S. aureus* biofilm cells and was concentration-dependent. When treated with 1 × MIC, LBA decreased the metabolic activity by 33.33%. The results indicated that LBA suppressed biofilm formation by interfering with *S. aureus* metabolic activity.

Hemolysins are the vital virulent elements in disease development [40]. α-toxin produced by *S. aureus* can lead to hemolysis as well as accelerate biofilm formation [41,42]. Hence, the anti-hemolytic effect of LBA on the RBC lysis of *S. aureus* was examined in liquid media. The results shown in Figure 4B were consistent with the observed antibiofilm activities; LBA suppressed the hemolytic activity of *S. aureus* and was concentration-dependent. There was no significant change at a low concentration of LBA at 0.125 × MIC. When treated with 0.25 × MIC, 0.5 × MIC, and 1 × MIC, LBA significantly suppressed the hemolytic activities with a decrease of 20.37%, 47.97%, and 70.21% (*p* < 0.01), respectively (Figure 4B). These results indicated that LBA inhibited the hemolysis activity of *S. aureus*. Further, the antibiofilm activity of LBA is associated with suppressing the hemolytic activity of *S. aureus*. It is reported that hemolysin production was critical for *S. aureus* virulence classified into four diverse types: alpha (α), beta (β), gamma (γ), and delta (δ). α-hemolysin secreted into the supernatant by *S. aureus* is a key factor that causes the hemolysis of RBCs. Some studies have stated that α-hemolysin can not only cause tissue damage and damage to the immune system but also affect the formation of biofilms [42] and activate the autophagy of *S. aureus* infection in host cells [43]. It also triggers secondary cellular responses such as eicosanoid production, cell release, and apoptosis [44]. Hence, the results indicated that LBA could suppress hemolysin activity and suppress virulence secretion, and the potential antibiofilm formation of *S. aureus* was associated with the anti-hemolytic and anti-virulence activities.

### 3.5. LBA Modulated Biofilm- and Virulence-Related Genes

**Figure 5:**
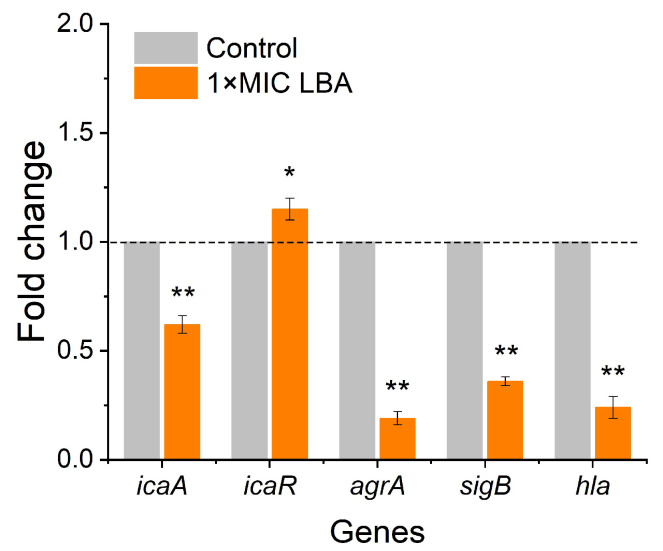
Expression of biofilm formation and virulence-related genes in *S. aureus* after treatment with 1 × MIC LBA by RT-qPCR. The expression level of 16s rRNA was used to normalize the expressions of the target genes. * *p* < 0.05, ** *p* < 0.01, compared with control.

To enhance the possible mechanistic insight into the antibiofilm of LBA against *S. aureus*, the expressions of five biofilm- and virulence-related genes in *S. aureus* at the transcription level were examined by qRT-PCR. From Figure 5, we can see the alters in the transcription levels of five biofilm- and virulence-related genes. When treated with 1 × MIC, LBA significantly (*p* < 0.01) decreased the expressions of the biofilm- and virulence-related genes *icaA*, *agrA*, *sigB*, and *hla* 1.64-fold, 5.26-fold, 2.78-fold, and 4.17-fold, respectively, while upregulating the biofilm-related gene *icaR* 1.15-fold (Figure 5). 

Acetyl-β-(1-6)-glucosamine (PIA/PNAG) is encoded by the *ica* operon and is crucial for the biofilm formation of *S. aureus* [45]. The intercellular adhesion (*ica*) locus consists of the *ica*ADBC operon which consists of the *icaA*, *icaD*, *icaC*, and *icaB* genes. *icaA* plays a vital role in producing polysaccharide intercellular adhesin and is needed for biofilm formation [46]. Notably, the fifth gene *icaR* is a negative regulator of *icaADBC* [47]. Additionally, the regulation of *ica*ADBC is complex and governed by diverse genes and regulatory factors such as *sigB*, a global transcriptional regulatory factor that was found to be a positive regulator of *ica*ADBC and might upregulate the expression of a factor involved in the production of PIA/PNAG [48]. In *S. aureus*, the auxiliary gene regulator *agr* is reported to be a major regulator of biofilm development and formation [48]. The Agr system consists of two units, RNAII and RNAIII, whose transcription depends on the activation of their respective *agr* promoters P2 and P3 [49]. Among them, RNAII (P2) contains four genes: *agrB*, *agrD*, *agrC*, and *agrA*. SigB negatively regulates *agr*, mainly by inhibiting the expression of RNA III, thereby reducing the activity of extracellular proteases and thus promoting the formation of biofilms. The virulence of *S. aureus* is attributed to various virulence factors, such as α-hemolysin encoded by *hla* [9], regulated under the control of the accessory gene regulator (*agr*) operon [50]. The constituent parts of the *agr* operon AgrA participate in driving the expression of diverse virulence factors and toxins in the post-exponential growth phase. Further, SigB also influences virulence factors’ production [51]. In the present study, LBA significantly downregulated the expressions of the biofilm- and virulence-related genes *icaA*, *agrA*, and *sigB* and upregulated the biofilm-related gene *icaR*, suggesting that these genes could be the key regulatory genes responsible for the inhibition of *S. aureus* biofilm formation and virulence by LBA, and provides a molecular explanation for the observed antibiofilm and anti-virulence activities of LBA.

Further, the *hla* gene is essential for the lethal α-hemolysis of *S. aureus* and contributes to biofilm formation [43]. LBA significantly (*p* < 0.01) repressed the expression of the α-hemolysin gene (*hla*) in *S. aureus* 4.17-fold (Figure 5), which was consistent with the formerly observed inhibition of hemolytic activity (Figure 4B). In the present study, LBA showed antibiofilm and antihemolytic activities that were consistent with previous studies that showed that tannic acid and trans-resveratrol both have antibiofilm activity and anti-hemolytic activity against *S. aureus* [52], which appears to a positive relation between antibiofilm and anti-hemolytic activities [53]. 

## 4. Conclusions

LBA exhibited potential inhibitory and inactivation properties against *S. aureus* biofilms and reduced the release of extracellular polymeric matrices in *S. aureus* biofilms, which led to the disruption of the biofilm architecture of *S. aureus* and loosened *S. aureus* microcolonies. Additionally, LBA repressed the metabolic activity and hemolytic activity of *S. aureus* and modulated the expression of biofilm- and virulence-related genes, thereby inhibiting the formation of *S. aureus* biofilm. These findings suggest that LBA has potential practical applications as a novel antibiofilm agent with potential anti-virulence properties to prevent and control *S. aureus* contamination and infections in food systems. LBA can be used with sanitizers or alone to be sprayed on the surfaces of food processing equipment for disinfection to prevent the spread of *S. aureus* at all stages of the food supply chain. Additionally, LBA could be used as an antibacterial packaging material in food storage and preservation to avoid the adhesion and retention of *S. aureus* on the surfaces of food and food packaging, controlling the spread of *S. aureus* and declining the occurrence of *S. aureus* contamination and infection to ensure food safety. 

## Figures and Tables

**Table 1:** Primer sequences of the targeted genes.

Genes	Primer Sequence (5’-3’)
*icaA*	forward: TTCCAGAAACATTGGGAGGTC reverse: CCTTTTCGTTTTCATTGTGCTA
*icaR*	forward: ACGCCTGAGGAATTTTCTGGA reverse: TTGCGAAAAGGATGCTTTCAA
*agrA*	forward: TCTCACAGACTCATTGCCCATT reverse: GGCGATTGACGACAAAGCT
*hla*	forward: GGTTTAGCCTGGCCTTCAGC reverse: ACCAGTAACATTACCGTTGAATCCA
*sigB*	forward: CTTTGAACGGAAGTTTGAAGCCT reverse: GCGGTTAGTTCATCGCTCACT
*16sRNA*	forward: ACTGGGCGTAAAGAGYTCGT reverse: CGCATTTCACCGCTACAC

## Data Availability

The original contributions presented in the study are included in the article, further inquiries can be directed to the corresponding author.

## References

[1] Pang X., Song X., Che M., Tian S., Lu Z., Sun J., Yuk H. (2022). Combating biofilms of foodborne pathogens with bacteriocins by lactic acid bacteria in the food industry. Compr. Rev. Food Sci. Food Saf..

[2] World Health Organization (2015). WHO Estimates of the Global Burden of Foodborne Diseases: Foodborne Disease Burden Epidemiology Reference Group.

[3] Arunachalam K., Pandurangan P., Shi C.L., Lagoa R. (2023). Regulation of *Staphylococcus aureus* Virulence and Application of Nanotherapeutics to Eradicate *S. aureus* Infection. Pharmaceutics.

[4] Suwal N., Subba R.K., Paudyal P., Khanal D.P., Koirala N. (2021). Antimicrobial and antibiofilm potential of curcuma longa linn. rhizome extract against biofilm producing *Staphylococcus aureus* and *Pseudomonas aeruginosa* isolates. Cell. Mol. Biol..

[5] Liu X., Yao H., Zhao X., Ge C. (2023). Biofilm Formation and Control of Foodborne Pathogenic Bacteria. Molecules.

[6] Guo A., Li Q., Liu L., Zhang X., Yao R. (2021). Formation of multi-species biofilms and their resistance to disinfectants in food processing environments: A review. J. Food Prot..

[7] Winkelstroter L., Teixeira F.B.D.R., Silva E.P., Alves V.F., De Martinis E.C.P. (2014). Unraveling microbial biofilms of importance for food microbiology. Microb. Ecol..

[8] Sun J., Wang D., Sun Z., Liu F., Du L., Wang D. (2021). The combination of ultrasound and chlorogenic acid to inactivate *Staphylococcus aureus* under planktonic, biofilm, and food systems. Ultrason. Sonochem..

[9] Chan W.C., Coyle B.J., Williams P. (2004). Virulence regulation and quorum sensing in staphylococcal infections: Competitive agrc antagonists as quorum sensing inhibitors. J. Med. Chem..

[10] Faleye O.O., Faleye O.S., Lee J.H. (2024). Antibacterial and antibiofilm activities of iodinated hydrocarbons against *Vibrio parahaemolyticus* and *Staphylococcus aureus*. Sci. Rep..

[11] Álvarez-Fernández E., Cancelo A., Díaz-Vega C., Capita R., Alonso-Calleja C. (2013). Antimicrobial resistance in *E. coli* isolates from conventionally and organically reared poultry: A comparison of agar disc diffusion and Sensi Test Gram-negative methods. Food Cont..

[12] Kiryu T., Kiso T., Nakano H., Ooe K., Kimura T., Murakami H. (2009). Involvement of Acetobacter orientalis in the production of lactobionic acid in Caucasian yogurt (“Caspian Sea yogurt”) in Japan. J. Dairy Sci..

[13] Alonso S., Rendueles M., Díaz M. (2013). Bio-production of lactobionic acid: Current status, applications and future prospects. Biotechnol. Adv..

[14] Kang S.M., Kong F.H., Shi X.Y., Han H.J., Li M.H., Guan B.Y., Yang M., Cao X.Y., Tao D.B., Zheng Y. (2020). Antibacterial activity and mechanism of lactobionic acid against Pseudomonas fluorescens and Methicillin-resistant *Staphylococcus aureus* and its application on whole milk. Food Control.

[15] Kang S.M., Kong F.H., Liang X.N., Li M.H., Yang N., Cao X.Y., Yang M., Tao D.B., Yue X.Q., Zheng Y. (2019). Label-free quantitative proteomics reveals the multitargeted antibacterial mechanisms of lactobionic acid against methicillin-resistant *Staphylococcus aureus* (MRSA) using SWATH-MS technology. J. Agric. Food Chem..

[16] Hou W., Kang S., Chang J., Tian X., Shi C. (2022). Correlation Analysis between GlpQ-Regulated Degradation of Wall Teichoic Acid and Biofilm Formation Triggered by Lactobionic Acid in *Staphylococcus aureus*. Foods.

[17] Zhang H., Li S., Cheng Y. (2022). Antibiofilm Activity of Allicin and Quercetin in Treating Biofilm-Associated Orthopaedics Infection. Appl. Biochem. Biotechnol..

[18] Shen F., Ge C., Yuan P. (2022). Metabolomics Study Reveals Inhibition and Metabolic Dysregulation in *Staphylococcus aureus* Planktonic Cells and Biofilms Induced by Carnosol. Front. Microbiol..

[19] Lyu X., Li C., Zhang J., Wang L., Jiang Q., Shui Y., Chen L., Luo Y., Xu X. (2021). A Novel Small Molecule, LCG-N25, Inhibits Oral Streptococcal Biofilm. Front. Microbiol..

[20] Rice K.C., Mann E.E., Endres J.L. (2007). The *cidA* murein hydrolase regulator contributes to DNA release and biofilm development in *Staphylococcus aureus*. Proc. Natl. Acad. Sci. USA.

[21] Sivaranjani M., Gowrishankar S., Kamaladevi A., Pandian S.K., Balamurugan K., Ravi A.V. (2016). Morin inhibits biofilm production and reduces the virulence of *Listeria monocytogene*–an in vitro and in vivo approach. Int. J. Food Microbiol..

[22] Lee J.H., Kim Y.G., Lee J. (2022). Inhibition of *Staphylococcus aureus* biofilm formation and virulence factor production by petroselinic acid and other unsaturated C18 fatty acids. Microbiol. Spectr..

[23] Kang S.M., Shi C.L., Chang J., Kong F.H., Li M.H., Guan B.Y., Zhang Z.H., Shi X.Y., Zhao H.W., Peng Y.Q. (2021). Label free-based proteomic analysis of the food spoiler *Pseudomonas fluorescens* response to lactobionic acid by SWATH-MS. Food Control.

[24] Akbas M.Y., Kokumer T. (2015). The prevention and removal of biofilm formation of *Staphylococcus aureus* strains isolated from raw milk samples by citric acid treatments. Int. J. Food Sci. Technol..

[25] Raja A.F., Ali F., Khan I.A., Shawl A.S., Taneja S.C. (2011). Antistaphylococcal and biofilm inhibitory activities of acetyl-11-keto-β-boswellic acid from Boswellia serrata. BMC Microbiol..

[26] Bai J.R., Zhong K., Wu Y.P., Elena G., Gao H. (2019). Antibiofilm activity of shikimic acid against *Staphylococcus aureus*. Food Control.

[27] Liu F., Du L.H., Zhao T., Zhao P., Doyle M.P. (2017). Effects of phenyllactic acid as sanitizing agent for inactivation of Listeria monocytogenes biofilms. Food Control.

[28] Fan Q.X., Yuan Y.H., Zhang T., Song W., Sheng Q.L., Yue T.L. (2022). Inhibitory effects of lactobionic acid on *Vibrio parahaemolyticus* planktonic cells and biofilms. Food Microbiol..

[29] Sharma S., Mohler J., Mahajan S.D., Schwartz S.A., Bruggemann L., Aalinkeel R. (2023). Microbial Biofilm: A Review on Formation, Infection, Antibiotic Resistance, Control Measures, and Innovative Treatment. Microorganisms.

[30] Mahto K.U., Priyadarshanee M., Samantaray D.P., Das S. (2022). Bacterial biofilm and extracellular polymeric substances in the treatment of environmental pollutants: Beyond the protective role in survivability. J. Clean. Prod..

[31] Fan Q.X., He Q., Zhang T., Song W., Sheng Q.L., Yuan Y.H., Yue T.L. (2022). Antibiofilm potential of lactobionic acid against *Salmonella* Typhimurium. LWT.

[32] Sivasubramanian S., Nizam M.N., Jeyaraj G.P., Shunmugiah K.P., Arumugam V.R. (2017). In vitro and in vivo exploration of palmitic acid from *Synechococcus elongatus* as an antibiofilm agent on the survival of *Artemia franciscana against* virulent vibrios. J. Invertebr. Patholo..

[33] Carla R.A., Davide C., Pietro S., Lucio M., John W.C. (2012). Biofilm formation in Staphylococcus implant infections. A review of molecular mechanisms and implications for biofilm-resistant materials. Biomaterials.

[34] Lu L., Hu W., Tian Z., Yuan D., Yi G., Zhou Y., Cheng Q., Zhu J., Li M. (2019). Developing natural products as potential anti-biofilm agents. Chin. Med..

[35] Valliammai A., Sethupathy S., Priya A., Selvaraj A., Bhaskar J.P., Krishnan V. (2019). 5-Dodecanolide interferes with biofilm formation and reduces the virulence of Methicillin-resistant *Staphylococcus aureus* (MRSA) through up regulation of agr system. Sci. Rep..

[36] Farha A., Yang Q., Kim G. (2020). Inhibition of multidrug-resistant foodborne *Staphylococcus aureus* biofilms by a natural terpenoid (+)-nootkatone and related molecular mechanism. Food Control..

[37] Das T., Sharma P.K., Busscher H.J., Mei H.C., Krom B.P. (2010). Role of extracellular DNA in initial bacterial adhesion and surface aggregation. Appl. Environ. Microbiol..

[38] Das T., Sehar S., Koop L. (2014). Influence of calcium in extracellular DNA mediated bacterial aggregation and biofilm formation. PLoS ONE.

[39] Khan S.N., Khan S., Iqbal J., Khan R., Khan A.U. (2017). Enhanced Killing and Antibiofilm Activity of Encapsulated Cinnamaldehyde against *Candida albicans*. Front Microbiol..

[40] Ariyanti D., Salasia S.L.O., Tato S. (2011). “Characterization of Haemolysin of *Staphylococcus Aureus* Isolated from Food of Animal Origin”, lndones. J. Biotechnol..

[41] Song L. (1996). Structure of staphylococcal α-hemolysin, a heptameric transmembrane pore. Science.

[42] Caiazza N.C., O’toole G.A. (2003). Alpha-toxin is required for biofilm formation by *Staphylococcus aureus*. J. Bacteriol..

[43] Mestre M.B., Fader C.M., Sola C., Colombo M.I. (2010). Alpha-hemolysin is required for the activation of the autophagic pathway in *Staphylococcus aureus*-infected cells. Autophagy.

[44] Smith-Palmer A., Stewart J., Fyfe L. (2004). Influence of subinhibitory concentrations of plant essential oils on the production of enterotoxins A and B and alpha-toxin by *Staphylococcus aureus*. J. Med. Microbiol..

[45] O’Gara J.P. (2007). *ica* and beyond: Biofilm mechanisms and regulation in *Staphylococcus epidermidis* and *Staphylococcus aureus*. FEMS Microbiol. Lett..

[46] Yu D., Zhao L., Xue T., Sun B. (2012). *Staphylococcus aureus* autoinducer-2 quorum sensing decreases biofilm formation in an *icaR*-dependent manner. BMC Microbiol..

[47] Cue D., Lei M.G., Lee C.Y. (2012). Genetic regulation of the intercellular adhesion locus in staphylococci. Front. Cell. Inf. Microbiol..

[48] Ikonomidis A., Vasdeki A., Kristo I., Maniatis A.N., Tsakris A., Malizos K.N. (2009). Association of biofilm formation and methicillin-resistance with accessory gene regulator (*agr*) loci in Greek *Staphylococcus aureus* clones. Microb. Pathog..

[49] Ji G., Beavis R.C., Novick R.P. (1995). Cell density control of staphylococcal virulence mediated by an octapeptide pheromone. Proc. Natl. Acad. Sci. USA.

[50] Mansson M., Nielsen A., Kjærulff L., Gotfredsen C.H., Wietz M., Ingmer H., Gram L., Larsen T.O. (2011). Inhibition of Virulence Gene Expression in *Staphylococcus aureus* by Novel Depsipeptides from a Marine *Photobacterium*. Mar. Drugs.

[51] Mitchell G., Fugère A., Pépin Gaudreau K., Brouillette E., Frost E.H., Cantin A.M., Malouin F. (2013). SigB is a dominant regulator of virulence in *Staphylococcus aureus* small-colony variants. PLoS ONE.

[52] Cho H.S., Lee J.H., Cho M.H., Lee J. (2015). Red wines and flavonoids diminish *Staphylococcus aureus* virulence with anti-biofilm and anti-hemolytic activities. Biofouling.

[53] Lee J.H., Kim Y.G., Yong R.S. (2016). Calcium-chelating alizarin and other anthraquinones inhibit biofilm formation and the hemolytic activity of *Staphylococcus aureus*. Sci. Rep..

